# Behavioural recovery following treatments for varicose veins: developing a measure of outcome and process

**DOI:** 10.1186/1745-6215-14-S1-O49

**Published:** 2013-11-29

**Authors:** Seonaidh Cotton, Jill Francis, Denise Bolsover, Maria Prior, Graeme MacLennan, Julie Brittenden

**Affiliations:** 1 University of Aberdeen, Aberdeen, UK; 2 City University London, London, UK

## 

The endpoints of recovery are frequently reported, but the process of recovery has received less attention. To investigate the process of recovery in the CLASS trial (Comparison of Laser, Surgery and foam Sclerotherapy) comparing treatments for varicose veins, we developed an instrument to assess patient-valued outcomes such as return to normal activities to identify potential modifiers of the effect of treatment.

To develop this instrument, we interviewed 17 patients who been treated for varicose veins (laser, surgery or foam sclerotherapy) 6-12 weeks previously. We used a topic guide to elicit personally significant behavioural milestones (eg driving, going out socially). All participants gave informed consent. Interviews were audio-taped and transcribed verbatim. Analysis involved highlighting each utterance that referred to a specific behaviour, developing labels to describe behaviours (eg bending the legs) and quantifying the number of times a particular behaviour was mentioned. In a Discriminant Content Validation exercise based on the WHO International Classification of Functioning, Disability and Health (ICF), behaviours were independently coded as Activity, participation, or both. The behaviours identified were used to generate questionnaire items.

Response scales were designed to assess both the relevance of each behaviour and how soon, after treatment, it was first performed. Preliminary versions of the questionnaire were piloted. Wording and formatting were refined in response to this testing.

The questionnaire has since been used to assess behavioural recovery in the CLASS trial and preliminary results will be presented. These methods could be used to develop other treatment-specific behavioural recovery questionnaires.

